# Knock-down of *odr-3* and *ife-2* additively extends lifespan and healthspan in *C. elegans*

**DOI:** 10.18632/aging.203518

**Published:** 2021-09-09

**Authors:** Ioan Valentin Matei, Vimbai Netsai Charity Samukange, Gabriela Bunu, Dmitri Toren, Simona Ghenea, Robi Tacutu

**Affiliations:** 1 Systems Biology of Aging Group, Institute of Biochemistry of the Romanian Academy, Bucharest, Romania; 2 The Shraga Segal Department of Microbiology, Immunology and Genetics, Center for Multidisciplinary Research on Aging, Ben-Gurion University of the Negev, Beer-Sheva, Israel

**Keywords:** lifespan extension, genetic interventions, synergism, ife-2, odr-3

## Abstract

Genetic manipulations can ameliorate the aging process and extend the lifespan of model organisms. The aim of this research was to identify novel genetic interventions that promote both lifespan and healthspan, by combining the effects of multiple longevity-associated gene inactivations in *C. elegans*. For this, the individual and combined effects of the *odr-3* mutation and of *ife-2* and *cku-70* knock-downs were studied, both in the wild type and *daf-16* mutant backgrounds. We found that besides increasing the lifespan of wild type animals, the knock-down of *ife-2* (starting at L4) also extends the lifespan and healthspan of long-lived *odr-3* mutants. In the *daf-16* background, *ife-2* and *odr-3* impairment exert opposing effects individually, while the *daf-16; odr-3; ife-2* deficient animals show a similar lifespan and healthspan as *daf-16*, suggesting that the *odr-3* and *ife-2* effector outcomes converge downstream of DAF-16. By contrast, *cku-70* knock-down did not extend the lifespan of single or double *odr-3; ife-2* inactivated animals, and was slightly deleterious to healthspan. In conclusion, we report that impairment of *odr-3* and *ife-2* increases lifespan and healthspan in an additive and synergistic manner, respectively, and that this result is not improved by further knocking-down *cku-70*.

## INTRODUCTION

The aging process might be defined by the progressive loss of viability and by an increase in fragility and vulnerability [1, 2]. This in turn, results in a huge health-related cost for the elderly and a dramatic growth in the mortality rate. Understanding the mechanisms underlying aging is one of the major biological and biomedical challenges of our society, and could result in high dividends if the society would gain the capacity to extend lifespan, and more importantly healthspan (i.e. the interval of healthy, productive life years) [3–5]. Although there is still much debate about the molecular causes of aging, the general consensus in the field is that aging is malleable, and studies in model organisms have already shown that aging can be manipulated by both genetic and environmental factors [6–8]. Up until now, more than 2,200 single-gene interventions have been reported to modulate lifespan in model organisms [9]. Most of these genes have been found through genetic interventions, including partial or full loss-of-function mutations, RNA-induced gene silencing, gene over-expression, and genetic polymorphisms, which were reported to promote longevity or cause a premature aging phenotype [9]. More importantly, it has been shown that a large part of these genes play a conserved role as longevity regulators across diverse taxa [10], and some of them even share similar gene expression levels in long-lived species [11, 12], overall suggesting that some of the reported longevity-associated interventions could have therapeutic implications even in humans.

The effect on the mean and/or maximum lifespan of the modified organisms ranges from very modest values (5-10% change) up to very high values, for well-established longevity-associated genes - for example, two-fold for *daf-2* in worms [13], six-fold for SIR2 in yeast [14], and even ten-fold for *age-1* in worms [15]. Genetic modifications have been identified even in mammals, albeit the observed effects so far seem to be smaller (up to a maximum of 50%) [9]. These works have significantly increased our knowledge about the genetics of aging and longevity in model organisms, and they should be followed by investigations into the effect of epistatic, or more precisely synergistic gene combinations on lifespan. This aspect, however, has been unfortunately less popular, mainly because the epistasis between longevity-associated genes, and between the pathways they are involved in, is complex and most often non-linear [16, 17], thus requiring much time and resources to be studied. In a recent paper, describing the SynergyAge database, we have defined three types of synergism, applied to the general case of N genetic interventions: 1) *full synergism*, in which lifespan values are known for all intermediary strains that contain any combination of the N interventions and the lifespan change for the n-mutant is greater than the sum of lifespan changes for any two intermediary k-mutant and (N-k)-mutant, 2) *simple synergism*, in which lifespan values are known for the final strain (N interventions) and for all single gene interventions, but not for all intermediary k-mutants, and in which the lifespan effect of the N-gene combination is greater than the sum of all the individual effects, and 3) *partially known synergism*, in which values are available only for an incrementally built model and for all genetic interventions in an N-sequence an increase in lifespan is observed [18].

The few seminal discoveries regarding longevity synergism generally include the well known IIF/FOXO pathway and the *daf-2*/*daf-16* genes, and have been started in *C. elegans* [6, 19, 20]. The SynergyAge database reports 62 synergistic combinations of pro-longevity interventions that include daf-2. Interestingly, based on SynergyAge data, we did not observe a general correlation between the strength of the longevity effect in WT with those in the long-lived *daf-2* mutant. For example, RNAi of *let-363* did not extend the lifespan of the *daf-2(mu150)* mutant [21], even though the two genes have the 2nd and 3rd largest increase of lifespan in WT (according to GenAge). *sod-2*, another important longevity-associated gene, whose deletion leads to a lifespan increase in WT, does not further extend the lifespan of *daf-2* mutants [22]. Moreover, three of the top *daf-2* enhancers have only a small effect in WT, when kept under same conditions as in the *daf-2* background: *clk-1* increases lifespan by only 1.18% compared to WT at 25° C [23] even though at this temperature extends *daf-2* lifespan by 205%; *rsks-1* increases lifespan of *daf-2* by 106%, but only by 20% in the WT [24]; *drp-1*, which potentiates the effect of *daf-2* by 73%, increases lifespan of WT by only 2% [25]; *clk-2* increases *daf-2* effect by +113% while in the WT the effect is limited to 68% [26]. In our study, the genes to be tested were selected based on several bioinformatic criteria (potential of being longevity enhancers for the *daf-2* knock-down, genes being part of individual clusters in a cross-database interactome, number of shared KEGG pathways, chromosome positions, etc.), followed by manual curation and evaluation (of scientific literature) for the short-listed gene combinations.

In mammals, the homologues of *daf-2* and *daf-16* are components of the mammalian insulin and insulin growth factor (IGF) signal transduction cascade (IIS) [27–29]. DAF-2 regulates endocrine responses to food availability, including longevity, dauer formation, and fat metabolism [13, 30, 31]. Mutations that reduce the function of DAF-2 extend lifespan through a mechanism that greatly depends on the activity of DAF-16 [32, 33]. In addition to the central role in integrating signals from insulin/insulin-like pathways, DAF-16 integrates signals from multiple upstream pathways to regulate various biological processes [34]. Due to the increased amount of data on *daf-2* and *daf-16* mutants, it is extremely appealing to search for genetic interventions that act synergistically amongst themselves, but also with the *daf-2* long-lived background. In this study, three such genes have been considered: *odr-3*, *ife-2* and *cku-70*.

Several sensory neurons are responsible for chemotaxis to volatile attractants found in food, pheromones or noxious odors [27, 35, 36], the nutrient perception by olfactory neurons being partially mediated by the DAF-2 pathway [36]. ODR-3, a G alpha protein with similarities to the members of Gi/Go protein family, is expressed in the sensory cilia of olfactory neurons, providing the main stimulatory signals for AWA and AWC sensory neurons [37, 38]. Ablation of AWA and AWC sensory neurons, as well as loss-of-function mutations in *odr-3*, extend lifespan through a pathway that depends partially or completely on signaling via DAF-16 [27, 36, 39]. Food restriction can promote an adaptive metabolic response such as mobilization of fat stores through activation of AWC neurons [40], and decreased DAF-2 signaling is known to affect cellular metabolism by promoting the accumulation of lipids in the intestine and hypodermis [30]. All these suggest a link between food sensing, metabolic adaptation and longevity. On the other hand, the *daf-2(e1370); odr-3(n1605)* double mutant shows a greater lifespan extension than either of the single mutants and even than their cumulative effects, thus *odr-3* and *daf-2* could also function through complementary pathways [39].

While the relationship between ROS and longevity is still not completely understood and ROS can have both beneficial or detrimental effects on lifespan, most of the genetic manipulations that decrease ROS lead to an increased lifespan [41]. Like its mammalian orthologue, eIF4E, the *C. elegans* IFE-2 plays an important role in protein synthesis and its inactivation protects against oxidative stress and extends lifespan [42]. Since *ife-2* impairment was found to extend the lifespan of long-lived mutants such as *daf-2*, *clk-1*, *eat-2* and *let-363*, it was suggested that down-regulation of protein synthesis induced by *ife-2* deficiency might represent a distinct mechanism by which lifespan is regulated [21, 42]. However, *ife-2* inactivation might extend lifespan not only by decreasing the rate of protein synthesis, but also by regulating mitochondrial and peroxisomal metabolism, which in turn, could stabilize the homeostasis of reactive oxygen species and increase cellular accumulation of trehalose [43].

Lastly, CKU-70 is the *C. elegans* orthologue of KU70, which in mammals participates with KU80 to the DNA repair of double-strand breaks [44]. Downregulation of CKU-70 activity was found to increase sensitivity to genotoxic stress and thermotolerance, thus indicating a conserved role in both DNA repair and stress response [45, 46]. Although RNA interference (RNAi) of *cku-70* increases the lifespan of wild type (WT) animals only in an RNAi sensitized background, the fact that *cku-70* knock-down extends the lifespan of *daf-2* mutants as well [46] suggests that *cku-70* might have an important role in aging.

Since *odr-3*, *ife-2* and *cku-70* deficiencies all potentiate the lifespan-extending effects of *daf-2* mutants, it is also interesting to find if their mechanisms involve downstream pathways that converge toward common effectors. In this work, we analyzed the effect of combined interventions in *odr-3*, *ife-2* and *cku-70*, on lifespan and healthspan, starting with L4 age. Since our lifespan and healthspan assays were carried out for all the combinations of the above-mentioned interventions, the use of synergism in the remainder of the paper refers to the "full synergism" definition. Our results show that simultaneous suppression of *odr-3* and *ife-2* functions additively extends lifespan and synergistically improves healthspan in a *daf-16* dependent manner. Knock-down of *cku-70* did not confer further benefits to lifespan or motility of *odr-3*; *ife-2* mutants.

## RESULTS

### RNA interference of *ife-2* but not *cku-70* increases lifespan of the long-lived *odr-3* mutants

**Figure 1:**
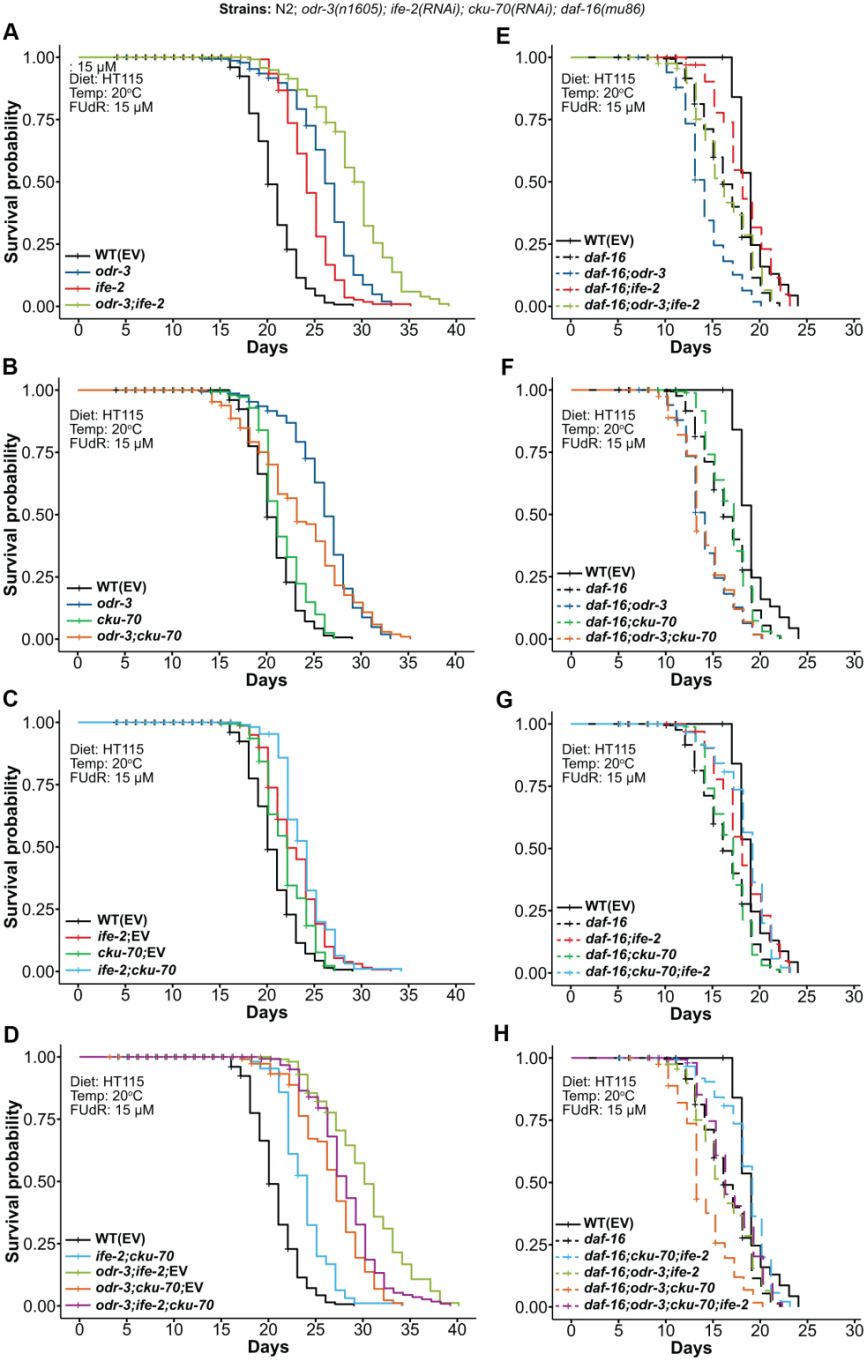
**Kaplan-Meier survival curves depicting the effects of combined genetic interventions on *odr-3*, *ife-2* and *cku-70* at 20° C.** (**A**–**D**) Lifespan comparisons in the WT background (continuous lines). (**E**–**H**) Lifespan comparisons in the *daf-16(mu86)* background (dashed lines). Survival curves represent: (**A**, **E**) *odr-3(n1605)* and *ife-2*(RNAi) single and double genetic interventions; (**B**, **F**) *odr-3(n1605)* and *cku-70*(RNAi) single and double genetic interventions; (**C**, **G**) *ife-2*(RNAi) and *cku-70*(RNAi) single and double genetic interventions; (**D**, **H**) *odr-3(n1605)*, *ife-2*(RNAi) and *cku-70(RNAi)* double and triple genetic interventions. (**C**, **D**) Control in the case of single RNAi knock-downs refers to treatment with a 1:1 mixture of RNAi bacteria and EV bacteria, in order to be comparable to the double RNAi intervention. (**A**–**H**) The survival plots in the WT background represent pooled populations from 3 independent experiments, whereas survival plots in the *daf-16(m28)* background represent pooled populations from 2 independent experiments. *odr-3* denotes *odr-3(n1605)* fed with EV; *daf-16* denotes *daf-16(mu86)* fed with EV; all strains in these experiments were grown on agar plates with *E. coli HT115(DE3)* and FUdR.

To find new potential genetic interactions that could extend lifespan, we assessed the effect of a simultaneous depletion of ODR-3, IFE-2 and CKU-70. For this, we used the *odr-3(n1605)* putative null allele [37] and we knocked-down *ife-2* and *cku-70* by RNAi. The *odr-3(n1605)* animals exhibited at 20° C an increased mean (26.2%) and maximum (13.8%) lifespan compared with WT control animals. A significant mean lifespan extension was previously reported for *odr-3(n1605)* at 25° C, however the increase was very modest at 20° C [39]. Silencing of *ife-2* by RNAi showed an 18.0% and 20.7% extension for the mean and maximum lifespan of WT, respectively (Figure 1A and Supplementary Table 1), which are in agreement with previously reported data for both *ife-2(ok306)* mutants and *ife-2* downregulated animals [21, 42]. In our experiments, the RNAi knock-down of *cku-70* in the WT worms produced only a marginal 4.4% increase for mean lifespan and was even slightly detrimental to maximum lifespan reducing it by 6.9% (Figure 1B and Supplementary Table 1), which is in agreement with previously reported data [46].

The *odr-3(n1605); ife-2*(RNAi) mutants exhibited more than 11% and 18% increase in mean lifespan compared with the *odr-3(n1605)* and *ife-2(RNAi)* single gene interventions, respectively (Table 1). Similarly, the maximum lifespan was also increased by more than 18% and 11% (Supplementary Table 1). Overall, compared with the WT controls, the combined *odr-3* and *ife-2* interventions extended mean and maximum lifespan by 40.3% and 34.5%, respectively (Figure 1A and Table 1 and Supplementary Table 1). This effect demonstrates an almost additive impact on mean lifespan, i.e. 40.3% increase compared to 44.2%, the sum of the two individual effects (Table 1). Similarly, the lifespan extension for *odr-3; ife-2; EV* (46.1%), i.e. worms exposed to a 1:1 mixture of HT115 (Empty Vector - EV) bacteria and of *ife-2* RNAi clone, was greater than the sum of individual effects (26.2% + 11.2%), supporting the existence of additive/synergistic mechanisms (Table 1).

**Table 1:** Mean lifespan of *C. elegans* strains with genetic interventions in the *odr-3*, *ife-2* and *cku-70*.

**Strain**	RNAi*	**Mean lifespan days ± SD**	**Effect vs control**	**p-value**		**Strain**	RNAi*	**Mean lifespan days ± SD**	**Effect vs control**	**p-value**
WT	EV	20.6±0.2	*[control]*			WT	EV	20.6±0.2	*[control]*	
*odr-3*		26.0±0.3	26.2 %	<2.0E-16		*odr-3*		26.0±0.3	26.2 %	<2.00E-16
WT	*ife-2*	24.3±0.2	18.0 %	<2.0E-16		WT	*ife-2;EV*	22.9±0.3	11.2 %	1.00E-10
WT	*cku-70*	21.5±0.2	4.4 %	7.00E-03		WT	*cku-70;EV*	21.9±0.2	6.3 %	1.00E-04
*odr-3*	*ife-2*	28.9±0.4	40.3 %	<2.0E-16		*odr-3*	*ife-2;EV*	30.1±0.5	46.1 %	<2.00E-16
*odr-3*	*cku-70*	23.4±0.5	13.6 %	4.00E-11		*odr-3*	*cku-70;EV*	26.5±0.4	28.6 %	<2.00E-16
						WT	*ife-2;cku-70*	23.7±0.3	15.0 %	3.00E-16
						*odr-3*	*ife-2;cku-70*	27.9±0.3	35.4 %	<2.00E-16

*ife-2 denotes animals fed only with *ife-2* RNAi bacteria, whereas *ife-2*;*EV* denotes animals fed with a mixture of ife-2RNAi/RNAi(EV). Similar for *cku-70*. *odr-3* denotes animals fed with RNAi(EV).

Next, we assessed the effect of *cku-70* silencing in both *odr-3(n1605)* mutant animals and *ife-2* knock-down animals. We observed that *cku-70* knock-down dramatically decreased the extension of mean lifespan conferred by the *odr-3(n1605)* mutation, from 26.2% to only 13.6% increase comparative with WT (Figure 1B and Supplementary Table 1).

The simultaneous knock-down of *ife-2; cku-70* by RNAi was performed by co-feeding worms with a mixture of the two RNAi bacterial clones. As such, for an appropriate comparison, the survival curves of double knock-down worms (which are presumably exposed to about half dsRNA for each gene) have been compared with those of single knock-down worms exposed to the same concentration of dsRNA for each of the corresponding genes (concentrations obtained by co-feeding the worms with the target RNAi clone and the control RNAi(EV) in a 1:1 ratio). In general, we obtained very small differences between the lifespan of worms fed only with the RNAi clone and worms fed with the mixture of RNAi clone / RNAi(EV) (Supplementary Figure 1A–1C), with the only notable difference being for *odr-3; cku-70* for which the mix (and hence lower concentration of *cku-70* RNAi bacteria) did not show a pronounced lifespan reduction (Supplementary Figure 1D).

In our assays, the lifespan of the double knock-down worms *ife-2; cku-70* was 15% longer than that of WT animals (p = 3.0E-16), with a small increase compared to each of the single knock-down worms (3.5% and 8.22% longer lived than the *ife-2*; EV and the *cku-70*; EV animals, respectively) (Figure 1C and Supplementary Table 1). It should however be noted that these changes are very modest and that a slightly larger lifespan increase was obtained when worms were exposed to *ife-2* RNAi alone, without EV mixing (18% compared to WT). Moreover, *cku-70* knock-down had a negative effect on the lifespan of *odr-3(n1605)* mutants treated with *ife-2* RNAi, decreasing the mean lifespan extension from 46.1% to 35.4% (Figure 1D and Supplementary Table 1).

### The extended longevity of *odr-3*; *ife-2* double intervention might be independent of DAF-16

The FOXO family transcription factor DAF-16 is a transducer of many pro-longevity signaling pathways [47], thus it was natural to inquire to what extent the longevity of *odr-3; ife-2* double inactivated animals require DAF-16. To answer this, we used the null *daf-16(mu86)* allele [32, 48] that affects coding of all DAF-16 isoforms, to generate *daf-16(mu86); odr-3(n1605)* double mutants, and carried out RNAi silencing assays for *ife-2* and *cku-70* in this strain.

We observed that the lifespan extension induced by the *odr-3(n1605)* mutation was not only suppressed by the *daf-16(mu86)* mutation, but also that the lifespan of the *daf-16(mu86); odr-3(n1605)* double mutants were even shorter than the lifespan of the *daf-16* mutants alone (Figure 1E; mean and maximum lifespan decreased by 14.6% and 9.1% compared to *daf-16*; mean and maximum lifespan decreased by 26.3% and 16.7% compared with WT). A slight decrease of *daf-16(mu86)* lifespan induced by the *odr-3(n1605)* mutation was also previously reported [39]. Silencing of *ife-2* on the other hand, extended the lifespan of *daf-16* mutants (Figure 1E; mean lifespan 9.8% greater), although the mean and maximum lifespan were not completely reverted to the lifespan values of the WT (mean and maximum lifespan 5.3% and 4.2% lower than WT, respectively). The lifespan of the triple *daf-16; odr-3; ife-2* inactivated animals did not significantly differ from *daf-16* single mutants. This could be partly explained by the fact that DAF-16 is one of the main transducers of signaling pathways modulated by ODR-3 and IFE-2 activity. To clarify this aspect we examined the nuclear translocation of DAF-16::GFP in *odr-3(n1605)*, *ife-2(RNAi)* and *odr-3(n1605); ife-2(RNAi)* animals, respectively (Supplementary Figure 2). Whereas *odr-3(n1605)* animals showed weak DAF-16::GFP nuclear accumulation in posterior intestinal cells, suggesting that ODR-3 could affect longevity partially through DAF-16 pathway, we did not observe consistent nuclear accumulation of DAF-16::GFP in *ife-2(RNAi)* and *odr-3(n1605); ife-2(RNAi)* animals. Therefore, ODR-3 and IFE-2 could affect lifespan by mediating parallel signaling pathways, which in the *daf-16* background have antagonistic effects - *odr-3* further decreasing lifespan and *ife-2* partially increasing the *daf-16(mu86)* lifespan.

In contrast to a previous study that reported a slight decrease of *daf-16(m26)* lifespan by *cku-70* knockdown at 25° C [46], in our experiments the lifespan of *daf-16; cku-70* at 20° C was similar to that of *daf-16* single mutants, and *cku-70* knock-down did not significantly influence the lifespan of *daf-16; odr-3*, nor of *daf-16*; *ife-2* mutants (Figure 1F, 1G). The quadruple *daf-16; odr-3; ife-2; cku-70* mutants exhibited a lifespan similar to that of *daf-16* single mutants (Figure 1H).

**Figure 2:**
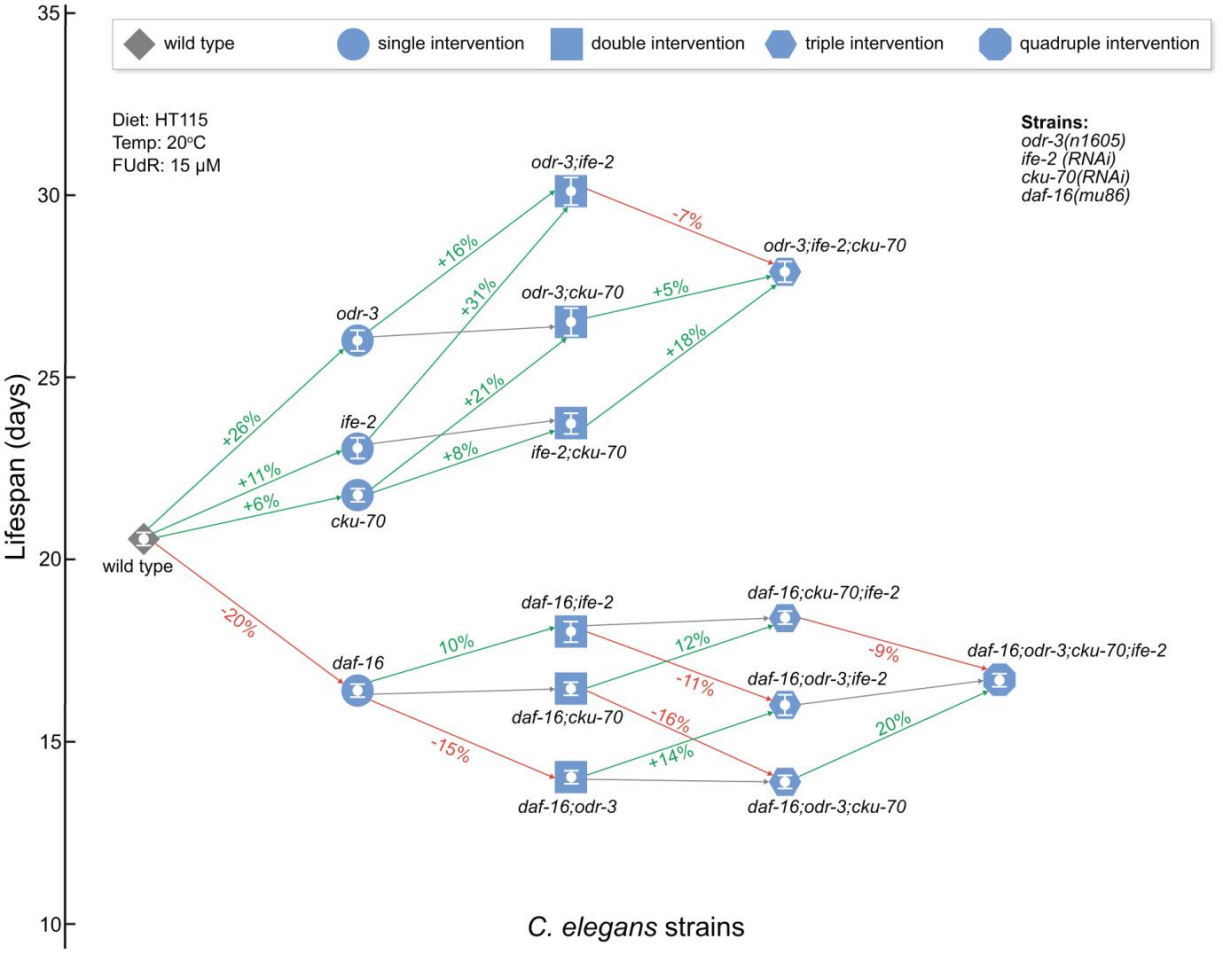
**Network schematic representation of the strains analyzed in this study and of the effects of each genetic intervention.** Nodes represent the strains as follows: diamond for WT, circle for single gene interventions, square for double gene interventions, hexagon for triple gene interventions, and octagon for quadruple gene interventions. Nodes are positioned on the vertical axis according to their respective mean lifespan. Edges between worm strains are colored depending on the gain (or loss) in lifespan extension: increase (green), decrease (red) and small or non-significant change (gray). The extent of the change is included on the edge as a percentage increase/decrease between the origin and destination nodes of the edge. *odr-3* and *daf-16* denote mutants containing the *odr-3(n1605)* and *daf-16(mu86)* mutations; *ife-2* and *cku-70* denote animals in which these genes were modulated by RNAi bacteria. The white bars inside of the nodes indicate the mean ± SEM.

Overall, our results show that simultaneous inactivation of *odr-3* and *ife-2* produce an additive lifespan effect, while the additional *cku-70* knock-down does not extend lifespan further (Figure 2). Since the lifespan effects observed are different in the *daf-16* background (Figure 2), it is possible that the mechanisms through which *odr-3; ife-2* animals achieve lifespan extension overlap with the pleiotropic mechanisms determined by *daf-16* (Figure 2).

### The *ife-2(ok306)* mutation also extends the lifespan of *odr-3(n1605)* mutant animals

**Figure 3:**
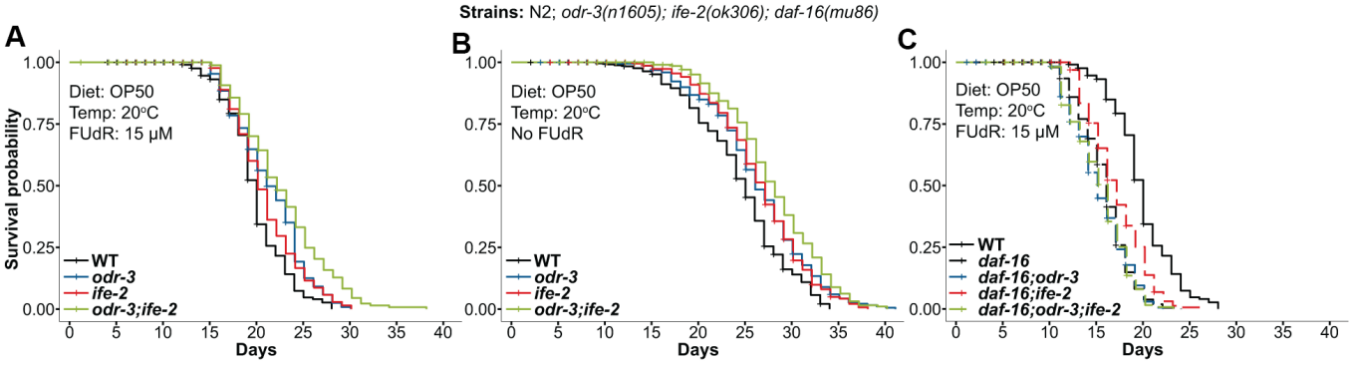
**Kaplan-Meier survival curves for animals containing the *odr-3(n1605)* and *ife-2(ok306)* mutations.** (**A**) *odr-3(n1605); ife-2(ok306)* single and double mutants, cultivated in the presence of FUdR. (**B**) *odr-3(n1605); ife-2(ok306)* single and double mutants, cultured without FUdR. (**C**) Lifespan comparisons for *odr-3* and *ife-2* in the *daf-16(mu86)* background. (**A**–**C**) Dashed lines are used for *odr*-*3(n1605)* and *ife-2(ok306)* mutants tested in the *daf16(mu86)* genetic background, while continuous lines are used for WT or single/double *odr-3* and *ife-2* mutants tested in the WT background. All cohorts were fed OP50 and kept at 20° C. All lifespan values can be viewed in the Supplementary Table 1.

One question is whether the *ife-2(ok306)* deletion mutation will produce similar effects as the *ife-2(RNAi)* in the *odr-3(n1605)* background. To answer this, we also generated the double *odr-3(n1605); ife-2(ok306)* and triple *daf-16(mu86)*; *odr-3(n1605); ife-2(ok306)* mutants and conducted further lifespan assays. The mutant worms were cultured at the same temperature (20° C) as in the RNAi experiments and were fed OP50 bacteria. We observed similar trends (Figure 3A), i.e. a 13.6% and 35.7% increase in mean and maximum lifespan for the *odr-3; ife-2* double mutant, compared to wild type, and an additive effect of the single mutations (the 13.6% increase in mean lifespan was comparable with the sum of the individual genetic effects of *odr-3* and *ife-2* mutations: 7.5% and 4.5%). It was however noticeable that in this experiment the impact of the double mutation on lifespan was smaller than in the case of *ife-2*(RNAi), which used an HT115 diet.

Since FUdR could cause an artefactual effect on the longevity of some mutants [49–51], we also conducted longevity experiments in the absence of FUdR. Similar to the previous results, we also observed an increased average lifespan for all strains even in the absence of FUdR (Figure 3B, *odr-3*:+8.2%, *ife-2*:+8.6% and *odr-3;ife-2*:+14.3%).

Next, we investigated the effect of the *odr-3(n1605)* and *ife-2(ok306)* mutations in the *daf-16(mu86)* background. Similar to the RNAi experiments, the *ok306* extended the lifespan of *daf-16* single mutants (Figure 3C, 6.25% increase), while the double *daf-16(mu86); odr-3(n1605)* and triple *daf-16(mu86); odr-3(n1605); ife-2(ok306)* mutants showed very similar lifespans as that of the *daf-16* (Figure 3C).

### The *odr-3; ife-2* impaired animals display increased motility and pharyngeal pumping

Our finding that RNAi impairment of *ife-2* in *odr-3(n1605)* animals increased lifespan prompted us to investigate the effect of their inactivation on healthspan. To assess healthspan, we focused on the evaluation of pharyngeal pumping and body movement. In *C. elegans*, these two physiological processes decrease with ageing, correlate between themselves and with other age-related declining properties, and ultimately can predict lifespan and healthy life [52].

**Figure 4:**
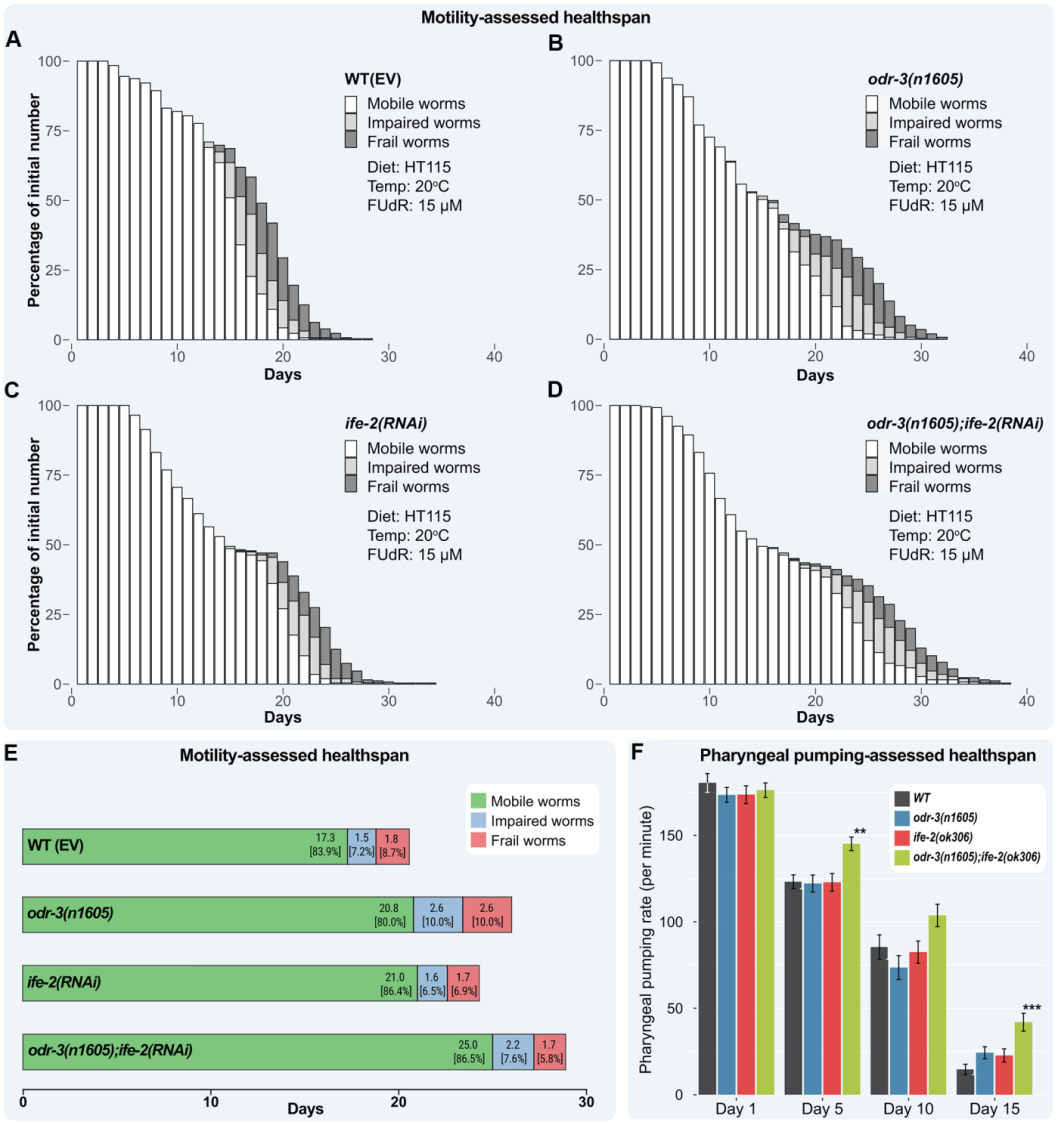
**Healthspan of combined genetic interventions on *odr-3* and *ife-2* at *20° C*.** (**A**–**D**) Bar chart representation of motility-assessed healthspan illustrating the fraction of each category upon daily monitorization. Worms are grouped into three categories: mobile (white), impaired (light gray) and frail (dark gray). Dead and censored animals were subtracted from these analyses. (**E**) Mean number of days in each motility state throughout lifespan. The mean time spent in the impaired state is computed as the difference between the mean time spent as mobile or impaired, and the mean time spent in the mobile state. The mean time spent in the frail state is computed as the difference between the mean lifespan and mean time spent as mobile or impaired. The values within brackets represent the distribution of motion stages during the lifespan. (**A**–**E**) WT(EV) and *odr-3* denote worms fed with RNAi(EV). (**C**, **D**) *ife-2* and *odr-3; ife-2* denote worms fed with *ife-2* RNAi bacteria. (**F**) The pharyngeal pumping rate (average number of contractions per minute) of WT, *odr-3(n1605)*, *ife-2(ok306)* and *odr-3(n1605); ife-2(ok306)* mutants were recorded on days 1, 5, 10 and 15 post-L4 moult. *odr-3(n1605); ife-2(ok306)* worms show a significantly slower decline of pharyngeal pumping with age, compared to WT. For simplicity, only significant differences among groups are indicated (one way ANOVA with Dunnett's test); ** denotes p < 0.01; *** denotes p < 0.001.

In our experiments, up to late adulthood, individual or joint interventions in *odr-3* and *ife-2* did not produce obvious pathological changes in the phenotype, indicating that the effect on locomotion was caused by physiological age-related changes, rather than a specific disease. As such, the observed motility status, carried out along the lifespan assay, can be viewed as a measure of the healthspan of the population. To quantify this, we classified individuals in three motion stages based on their ability to move. Stage A (healthy, fully mobile worms) included animals in a physiological state that could move without any impediment, stage B (impaired worms) included animals with diminished locomotion, whereas stage C (frail worms) included animals found in a frailty state. Animals were scored daily and associated with one of the motion stages. The distribution of stages for each strain is presented graphically in Figure 4A–4D (for motility data of *odr-3(n1605); ife-2(ok306)* mutants, see Supplementary Figure 3A–3H; for the *daf-16(mu86); odr-3(n1605); ife-2(ok306)* mutants see Supplementary Figure 3I–3L).

Considering the average healthspan of worms in stage A, we observed a similar trend to lifespan. Simultaneous inactivation of *odr-3* and *ife-2* produced a synergistic healthspan effect, while the additional *cku-70* knock-down does not extend healthspan further (Supplementary Figure 4).

Using the Kaplan-Meier method to estimate the fraction of mobile worms at each observation point, plotted against time, the average number of days the worms spent in each state was computed (Figure 4E and Supplementary Table 2). This allowed us to model the transitions from the healthy to impaired or frail (see Materials and methods), thus, determining if a change in the locomotion status was induced by the genetic interventions and whether in addition to lifespan, the ratio between healthspan and lifespan was also changed.

The WT animals spent on average 17.3 days in the mobile stage, which represent 84% of their mean lifetime, 1.5 days (7.3%) in an impaired stage and 1.8 days (8.7%) in the frailty stage. The *odr-3* mutants remained mobile longer than WT animals (on average 20.8 days), however relative to their mean lifespan, they were fully mobile only for 80.0% of their lifetime, thus exhibiting a proportional (or greater) lifespan fraction in which they were impaired or frail (2.6 days, 10.0%, for both). *ife-2* RNAi treated animals remained mobile on average more days (21.0 days, 86.4%) and exhibited a similar number of impaired (1.6 days, 6.6%) and frail days (1.7 days, 7.0%) (Supplementary Table 2).

For the *odr-3; ife-2* animals, the longest-lived strain in our study, a corresponding increase of days with full (25.0 days, 86.5%) and impaired motility (2.2 days, 7.6%) was observed, while the number of days of frailty remained similar as for WT animals (1.7 days, 5.9%).

Next, we compared each fraction of being fully mobile with the corresponding fraction in the WT animals to find if the genetic interventions indeed conferred significant benefits for the quality of life, i.e. increasing the fraction of time spent in a mobile state and decreasing the impaired and frailty fractions. By doing this, we found that although the *odr-3* mutants had extended longevity, this was not associated with a motility-based health benefit since the lifespan fraction in which worms lived as fully mobile actually decreased slightly by 4.8% (Supplementary Table 2), whereas the fraction of time spent in both the impaired and frailty stages increased by almost 40%. By contrast, *ife-2* RNAi treated animals stayed mobile for a similar lifespan fraction as WT animals (3% longer), but they spent much less time as impaired or frail (these fractions were 9.6% and 19.5%, respectively, smaller than those for WT). Compared to WT control animals, silencing of *ife-2* in *odr-3* worms did not affect the mean lifespan fraction spent in the mobile and impaired stages, but decreased the lifespan fraction for the frailty period by more than 30%.

To assess the significance of the changes in motility status, the Kaplan-Meier curves modeling the transitions from mobile to impaired and frailed were used and the relevant comparisons are included in the Supplementary Figure 5C. Overall, our findings show that the *ife-2* RNAi treatment and the double intervention *odr-3(n1605); ife-2(RNAi)* have a beneficial effect on motility-assessed healthspan, significantly increasing the period of full motility (Supplementary Figure 5C; p < 2.0E-16) and exhibiting a decrease in the decrepit period of life. Similar data for the *odr-3(n1605); ife-2(ok306)* double mutant can be seen in Supplementary Figure 5D, 5E and for the *daf-16(mu86); odr-3(n1605); ife-2(ok306)* triple mutant in Supplementary Figure 5F.

To further determine whether healthspan is affected, we analyzed the decline of pharyngeal pumping with age, a process controlled by the cardiac-like pharyngeal muscle. As seen in Figure 4F, the number of pharyngeal movements strongly decreases with age in all cohorts. The changes for both single mutants, *odr-3(n1605)* and *ife-2(ok306)*, are similar to those in the WT (no statistically significant difference observed when comparing to WT, at each day; one-way ANOVA). The *odr-3(n1605); ife-2(ok306)* double mutant showed a slower decline in pharyngeal contractions and a higher rate of pumping compared to WT. The improved healthspan was observed starting from day 5, when the average number of contractions was 17.8% higher than for WT (p = 0.002), and slightly increased, at day 10 being 21.4% higher (p = 0.14; ns). At day 15, the *odr-3;ife-2* animals still showed approximately 42 contractions per minute (compared to 15 contractions per minute in the WT), a 187% increase (p <E-04). On day 15, the *odr-3(n1605)* and *ife-2(ok306)* mutations showed a synergistic effect on the pharyngeal pumping, the increase observed in the double mutant being higher than the sum of the individual effects (187% increase compared to 66% + 55% increase for single mutants), corresponding to a fully synergic effect [18].

### The *odr-3; ife-2* double mutant displays an increased resistance to stress

**Figure 5:**
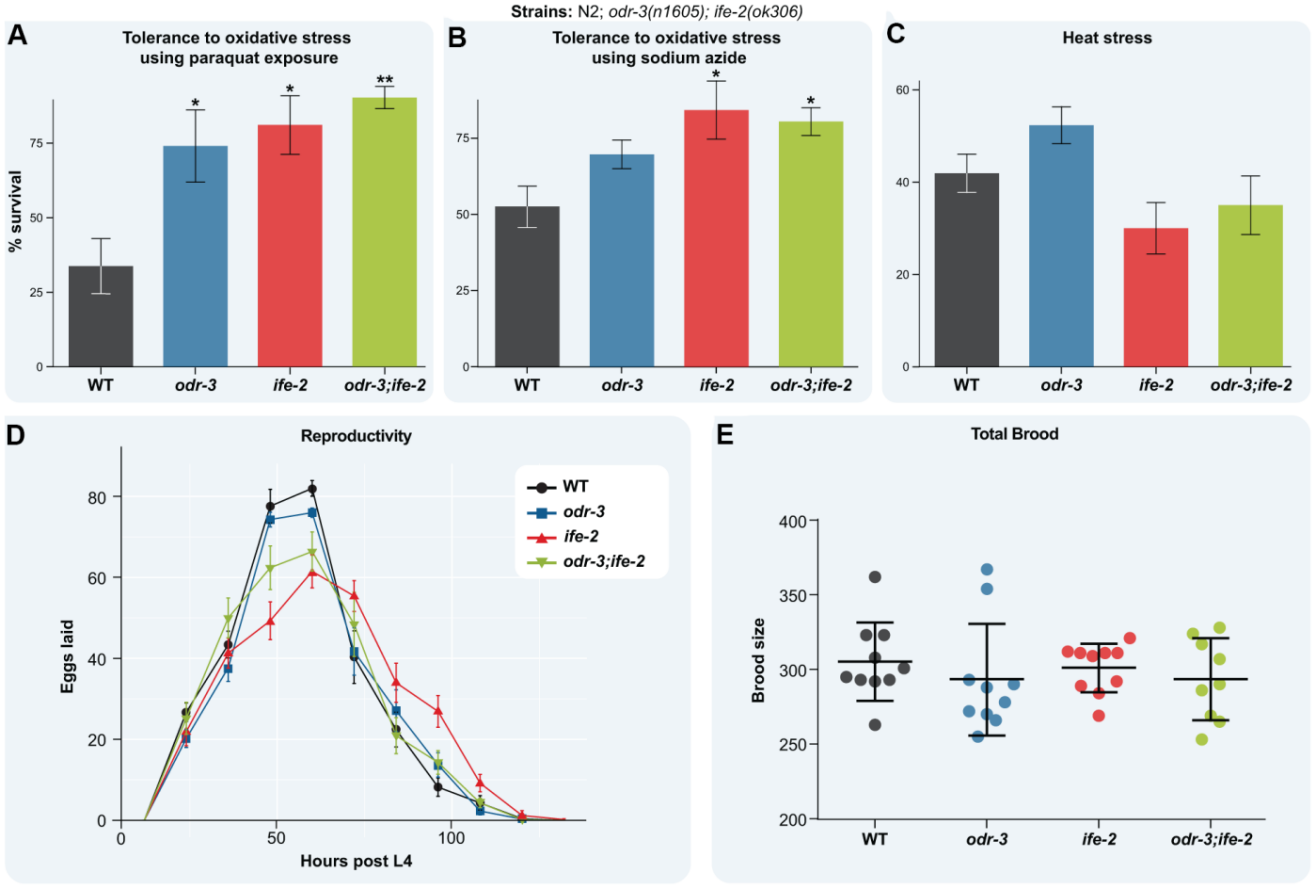
**Loss of *odr-3* and *ife-2* activity enhances oxidative stress tolerance.** (**A**) Survival fraction of the indicated L4 larvae upon 5 days exposure to 0.2M paraquat (the experiment was repeated independently three times). (**B**) Survival fraction of the indicated strains upon 1 hour treatment with 0.5M NaN3 (the experiment was repeated independently four times). (**C**) Survival fraction of the indicated strains upon 4h heat stress at 35° C. Each strain was scored on three replicate plates and the experiment was repeated independently four times. (**D**) Egg-laying rate of the indicated strains. The average number of eggs laid by each strain was determined by transferring worms to new *E. coli* plates every 12 hours from L4 stage. (**E**) Brood size of the indicated strains at 20° C. Each point represents the total brood of one hermaphrodite. (**A**–**E**) Bars indicate the mean ± SEM. For simplicity, only significant differences among groups are indicated (one way ANOVA with Dunnett's test); * denotes p < 0.05; ** denotes p < 0.01.

Resistance to stress declines with age after early adulthood [53], and the loss of protein homeostasis together with the failure to activate cellular stress responses are among the earliest aging marks [54]. Elevated temperatures perturb the protein homeostasis due to accumulation of defective proteins, whereas production of reactive oxygen species (ROS) and H_2_O_2_ increase global cellular damage. Both insults activate the cellular stress responses, aiming to improve cellular fitness and organismal recovery. To find if the double intervention in *odr-3 and ife-2* perturbs the stress response mechanisms, we monitored survival upon exposure to oxidative stress and acute heat stress. We induced oxidative stress by treatment with either paraquat, which generates reactive oxygen species, or NaN_3_, which generates H_2_O_2_. Both treatments dramatically reduce survival of WT animals by more than 50% (Figure 5A, 5B). In contrast, mutation in the *odr-3* increases survival of the animals treated with both paraquat and NaN_3_ compared with treated WT, although not statistically significant for NaN_3_. *ife-2* mutants exhibited an increased survival upon both treatments, as previously reported [42]. The *odr-3; ife-2* double mutants were less sensitive to ROS and H_2_O_2_ toxicity than WT animals, but they did not show an additive effect when compared with single mutants (Figure 5A, 5B).

Exposure to 35° C for 4h decreases the survival rate of WT animals by more than 50% (Figure 5C). Whereas the heat shock slightly increased the survival of *odr-3* mutants, compared with WT (not statistically significant), it did not also affect the survival of *ife-2* mutants or *odr-3; ife-2* double mutants (Figure 5C), overall indicating that impairment of *odr-3* and/or *ife-2* do not affect the heat stress response.

Some genetic or non-genetic interventions that extend lifespan also reduce fecundity, implying a trade-off between longevity and reproduction [55–57]. We did not observe such an effect for *odr-3* and *ife-2* single and double mutants (Figure 5D). Thus, the reproductive period and the age of the peak egg-laying rate of single and double mutants coincided with that of WT animals (Figure 5D). Moreover, the brood size of the mutants is similar to that of WT animals (Figure 5E). In conclusion, simultaneous depletion of *odr-3* and *ife-2* extends lifespan without affecting fecundity, promotes muscle activity and maintains activation of stress response mechanisms, consistent with increased health.

## DISCUSSION

Longevity is regulated by a combination of genetic and non-genetic factors, such as environmental interactions and lifestyle. The identification of genetic mutations that extend lifespan in model organisms has shown that longevity is mainly regulated by a complex interplay between many signaling pathways that affect cellular functions as diverse as nutrient sensing, genome stability, mitochondria fitness, organelle proteostasis, intercellular communication, transcription, proliferation and cellular regeneration [58, 59]. Previously, we have successfully used network-based approaches, building upon the list of known longevity-associated genes hosted in the GenAge database [9], to predict novel genetic or drug interventions that extend lifespan [60, 61]. However, due to the existence of complex and intricate interactions between hundreds of longevity-associated genes [62–64], the lifespan modulation obtained with these methods was limited by how much a single gene can influence longevity. While combined genetic interventions that modulate longevity *via* parallel pathways or drugs targeting multiple evolutionary conserved aging pathways have been shown in many instances to extend lifespan [65], the number of gene combinations tested so far has not been very high [18]. Here, we assessed the effects on both lifespan and healthspan, given by the simultaneous inactivation of three genes: *odr-3*, *ife-2* and *cku-70*. By reporting on the synergy of the pro-longevity effects of IFE-2 and ODR-3, and at the same time on the lack of synergy between CKU-70 and the above two genes, we hope that the current work will add to the accumulating data on longevity-related gene combinations, which could be used in future predictions of complex, multi-gene interventions.

According to the SynergyAge database - http://synergyage.info/ [18], the above-mentioned genetic interventions were among the most promising in terms of lifespan extension, when combined with IIS-defective *daf-2* mutants [39, 42, 46], a highly desirable property. SynergyAge hosts 133 unique synergistic interactions, involving 108 genes and of these, 62 gene combinations include daf-2. Much less combinations are antagonistic (32 gene combinations) while for 156 gene combinations the effect is somewhere between the two individual effects. Although this summary does not provide information on the number of negative results (which would probably be classified in one of the last 2 categories) it gives a sense of the scarcity of synergistic interactions discovered so far - considering the total number of potential gene combinations that could encompass for example the 889 worm longevity-associated genes from GenAge [9] (even for two gene combinations).

In *C. elegans*, ODR-3, IFE-2 and CKU-70 have different functions, and although inactivation of each of them extends lifespan, the mechanisms by which this occurs are at least partially different from each other. In our study, the lifespan and healthspan assays were carried out for all these 3 genes and their combinations. As a result, we obtained a clear perspective of the lifespan and healthspan changes from all worm strains, which is represented in Figure 2 and Supplementary Figure 4, respectively. Using this representation, it can be easily observed that the *odr-3; ife-2* double inactivation leads to an additive increase (i.e. the effect of the joint interventions is equal to the sum of the individual effects) in lifespan compared with single gene inactivations (Figure 2), as well as a synergistic effect on healthspan (Supplementary Figure 4). On the other hand, *cku-70* down regulation does not significantly affect the lifespan of *ife-2* mutants (Figure 1C) and is detrimental to the long-lived *odr-3* and *odr-3; ife-2* strains (Figure 1B, 1D). This detrimental effect is not seen in the *daf-16* mutants, where the loss of both *odr-3* and *daf-16* seems to be dominant compared to the influence of *cku-70* downregulation (Figure 1F). It is difficult to explain the effect of *cku-70* down regulation on *odr-3* and *odr-3; ife-2* only through its function in the DNA repair process. In addition to its role in DNA repair, the conserved Ku heterodimer was found to participate in other cellular processes such as transcriptional regulation, apoptosis, DNA replication, RNA metabolism and other [66, 67]. Therefore, a more complex interaction between *cku-70*, *odr-3* and *ife-2* might exist.

In our experiments we double inactivated *ife-2* and *cku-70* by RNAi. This raises the possibility that the knockdown efficiency of one or both genes could be reduced to half. For this reason, for an appropriate comparison with single RNAi down regulation we exposed the worms to a half concentration of dsRNA by mixing RNAi clone with EV clone. Unfortunately, we could not verify by quantitative PCR the efficiency of *ife-2* RNAi down regulation, which gave us the most notable effect, because the DNA fragment used for expression of dsRNA from L4440 vector encompasses the full gene (ORF and UTR) and due to the uptake of dsRNA in the cells, the endogenous *ife-2* mRNA cannot be distinguished and targeted for PCR amplification. However, knock down of *ife-2* RNAi; EV increased the lifespan of *odr-3* mutants, suggesting efficient down regulation even with half concentration of *ife-2* dsRNA. We have to point out that in the case of double RNAi there is a possibility that worms are not equally exposed to both dsRNA, and down regulation of one or both genes might be less efficient. Hence, although single downregulation of *cku-70* RNAi gave only a very modest increase of lifespan, the effect of double inactivation *ife-2(RNAi; cku-70(RNAi)* should be considered with care.

The increased lifespan of *odr-3; ife-2* might be simply explained by the combined effect of two genes acting in distinct pathways. However, since the *daf-16* mutation is epistatic to the *odr-3* mutation and greatly reduces the lifespan of *ife-2* knock-down animals, a more complex interaction is also possible. ODR-3 is expressed in 5 pairs of sensory neurons. In the AWA and AWC neurons, ODR-3 functions in perception and transduction of odor signals [37], in ADF and ASH mediates gustatory plasticity and detection of nociceptive stimuli, respectively [37, 68], whereas in AWC neurons is involved in temperature sensing [69]. Among these neurons, only AWA and AWC neurons were found to modulate lifespan [36], therefore it is thought that ODR-3 regulates longevity by functioning in these neurons. However, ODR-3 has a role in modulating the adaptive behavior to different stimuli by adjusting the levels of second messenger cGMP in response to environmental cues. We found that WT *C. elegans* grown on OP50 and HT115 diets have similar lifespans (20.6 mean lifespan on HT115 vs. 19.9 mean lifespan on OP50), indicating an efficient adaptive response of WT animals to these bacterial diets, as previously reported [70, 71]. In contrast, *odr-3(n1605)* mutants have an increased lifespan on HT115 diet (26% increase in mean lifespan on HT115 diet, comparative to 7.5% increase of mean lifespan on OP50 diet), which implicates ODR-3 in metabolic adaptation as it was found in the case of other metabolic genes [70, 71]. IFE-2 is expressed in all soma cells, including neurons [42]. The most pronounced lifespan and healthspan extension of *odr-3; ife-2* animals was observed when *ife-2* was down regulated by RNAi. Since RNAi interference is known to affect all tissues of the WT animals with the exception of neurons, these findings raise the possibility that non-neuronal silencing of *ife-2* by RNAi might be primarily responsible for the improved healthspan and extended longevity of the *odr-3; ife-2(RNAi)* animals. Alternatively, impaired protein synthesis in neurons due to *ife-2* deficiency might be detrimental to nematode health by affecting neuronal proteostasis [72]. Growing evidence unravels an important role for cell non-autonomous regulation of proteostasis in aging in which neuronal activation of stress response pathways such as heat shock response, mitochondrial and ER unfolded protein responses regulate nematode longevity by modulating cellular proteostasis in distal cells [73, 74].

DAF-16 stabilizes the transcriptome against the proteostasis collapse during aging by controlling the activity of hundreds of genes, integrating inputs from the DAF-2 pathway and from pathways that appear to regulate lifespan independently of DAF-2 [34, 75]. Therefore, the genetic interaction between *odr-3*, *ife-2* and *daf-16* could take many forms. In our experiments, although *ife-2* inactivation increased the lifespan of *daf-16(mu86)* mutants, a result that is in accordance with a previous report [42], it did not extend the lifespan beyond that of WT controls. We found that in contrast to *odr-3* mutation, which weakly activates DAF-16 in posterior intestine, down regulation of *ife-2* does not induce DAF-16 nuclear translocalization, implying that DAF-16 activity is not directly modulated by IFE-2. Both DAF-16 and IFE-2 could affect common processes such as metabolic remodeling and maintenance of cellular proteostasis that modulate longevity. Several metabolic changes were identified as fingerprints for long-lived mutants including the shift from carbon to amino acid catabolism as an alternative energy source, upregulation of lipid storage, increased purine metabolism and increased trehalose stores [43, 76, 77]. Many of these processes were found to be regulated in a DAF-16-dependent manner [75, 78–81]. In *mev-1* mutants, which lack succinate dehydrogenase cytochrome b, depletion of *ife-2* induces stress resistance but also restores WT lifespan [42]. A metabolomic study revealed that *ife-2* deficiency does not revert the mitochondrial *mev-1* defects, but rather restores the catabolism of purine nucleotides (e.g. GMP and AMP) and the metabolism of very long-chain fatty acids (VLFA) [82], processes related to peroxisomes. Since beta-oxidation of VLFA is a source of reactive oxygen species, and peroxisomes are sensitive to increased oxidative stress, *ife-2* depletion could also protect peroxisomes from oxidative stress, hence ameliorating peroxisomal function.

We found that the *odr-3; ife-2* double mutants are less sensitive to induced ROS or H_2_O_2_, however a relationship between this and the additive/synergistic nature of the combined intervention cannot be directly inferred. First, it was previously shown that stress resistance and lifespan can be experimentally dissociated and the magnitudes of changes in these two parameters produced by mutations are not identical [83]. Second, while it might be intuitive to suggest that the lack of additivity in the oxidative stress defence could mean the two genetic interventions activate the same mechanism, this is highly speculative, and small added differences in stress resistance could in fact affect longevity non linearly.

We also found that the decrease of motility and pharyngeal pumping, which decline in an age-related manner, were delayed in the *odr-3; ife-2* mutants. As seen in Figure 4E, the time spent by *odr-3; ife-2* animals (in absolute values) in a frail state does not increase, although their lifespan increases compared to both WT and single mutants. Together with the fact that the double intervention extends both mean and maximum lifespan (Supplementary Table 1), it suggests that animals remain healthy for a longer period, while the physiological decline that occurs during the advanced stage of aging is seemingly unaffected. Using an analogy to the socio-economic implications in a human population (if such an intervention could be translatable), such a therapy would probably not reduce the healthcare costs during late senescence, however it would increase the Healthy Life Years (HLY) indicator, which is a measure of productivity during life and an important economic factor.

Among the three genes that we investigated, the role of *ife-2* in aging was the most comprehensively analyzed, so far. Thus, it was shown that the long-lived mutants, *daf-2*, *age-1*, *let-363*, *clk-1*, *eat-2*, dramatically extended the lifespan of *ife-2* impaired animals [21, 42]. There is limited information about interaction of *odr-3* or *cku-70* with other long-lived mutants. Both, *odr-3(n1605)* and *cku-70RNAi* extended the lifespan of *daf-2(e1370)* mutants [39, 46]. We found that mutation in *odr-3* extended the healthspan of *ife-2* downregulated animals with a higher magnitude than it extended *ife-2* lifespan**,** suggesting that the effect of *odr-3* and *ife-2* impairment may not be due to a role of these genes in the control of longevity per se, but rather a consequence of a longer healthspan due to amelioration of age-related decline of physiological processes. This is supported by the observation that in contrast to *eat-2* mutants which have reduced pharyngeal pumping, the *odr-3; ife-2* animals exhibit a delay in the pharyngeal pumping decline, in older animals.

While much more work is probably needed to fully explore the mechanistic way in which the interaction between *odr-3* and *ife-2* modulates longevity, our results show that the knock-down of both *odr-3* and *ife-2* increases resistance to some types of stress and additively extends lifespan and healthspan.

## MATERIALS AND METHODS

### Strains and culture conditions

The following strains used in this study were provided by the Caenorhabditis Genetic Center (CGC): *C. elegans* wild-type Bristol strain (N2), CX3222 *odr-3(n1605)V*, RB579 *ife-2(ok306)*, CF1038 *daf-16(mu86)I,* OH16024 *daf-16(ot971 [daf-16::GFP])I, E. coli OP50* and *HT115(DE3)* strains. The *C. elegans* strains were maintained at 20° C using standard methods [84].

Multiple mutants were obtained by standard genetic methods and the presence of mutations was tested either by screening for characteristic phenotypes or via PCR genotyping. The homozygous *odr-3(n1605)* allele was confirmed by negative chemotaxis tests to isoamyl alcohol. To confirm the presence of homozygous *daf-16(mu86)* allele, high density populations were allowed dauer formation and subsequently tested for resistance to SDS 1%. Presence of *ife-2(ok306)* deletion was confirmed by PCR genotyping.

### *ife-2* and *cku-70* RNAi

For the RNAi-mediated gene knock-down by feeding method, a slightly modified protocol of the Ahringer technique was used [85]. Briefly, bacteria were grown overnight in LB medium supplemented with 50 μg/ml ampicillin and seeded onto NGM plates supplemented with 25 μg/ml carbenicillin and 1 mM IPTG. The plates were kept at room temperature for two days before use. Several L4 hermaphrodites picked from plates seeded with OP50 were placed onto RNAi plates, transferred the next day to other fresh RNAi plates, allowed to lay eggs for 24 h, then removed. The L4 hermaphrodites developed from eggs laid onto RNAi plates were used for longevity and healthspan assays. For double RNAi experiments, the plates were prepared in a similar way, with the exception that the plates were seeded with a 1:1 mixture of both RNAi bacterial clones. The *ife-2* and *cku-70* RNAi clones were obtained from the Ahringer RNAi library (Source BioScience, Nottingham, UK); both clones were validated by sequencing. The HT115 bacteria transformed with the L4440 empty vector, HT115 (EV), was used as control for RNAi experiments unless otherwise specified. When *ife-2; cku-70* double RNAi was employed, the control worms were grown on plates seeded with a 1:1 density mixture of HT115 (EV) bacteria and *ife-2* or *cku-70* RNAi clone, respectively, to maintain the same concentration of each double strand RNA as in the strains subjected to double RNAi.

### Lifespan assays

Since the age at which a treatment is started can significantly influence the outcome [86], all worm cohorts used in this work have been age-synchronized (L4 larvae stage). For all RNAi experiments, age-synchronized L4 larvae were manually transferred to RNAi agar plates containing 15 μM 5-fluorodeoxyuridine (FUdR). For lifespan assays of mutant animals, age-synchronized L4 larvae were manually transferred to NGM plates, and seeded with OP50. In case of lifespan assays without FUdR, the worms were transferred to a new plate every day until they ceased laying eggs, then when needed. For mutant animals cultured with FUdR, a 15 μM FUdR concentration (same as in the RNAi experiments) was used. In all cases, worms were kept at 20° C and scored daily as dead or alive based on their response to a gentle touch with a wire. Worms that presented externalization of internal organs, died because of bagging, or crawled up the wall of the dish were censored. For RNAi experiments, the WT control and *odr-3* animals were fed with HT115 (EV) bacteria. 85 worms were assayed per experiment.

While the lifespan assays were not conducted in a blinded manner, as suggested by Gruber et al., [87], the experiments were carried out by 3 operators, working with the data independently and results were evaluated for consistency. From the beginning of the study, all operators aimed to treat worm cohorts in an unbiased fashion and keep them in the same conditions.

### Locomotion assay

Animals were scored for free movement and for a response to prodding with a wire, daily, during the lifespan assay until death. Worms were classified into three motion stages, based on ability to engage and coordinate the body wall muscle in a forward or backward movement according to a previously described method [88], with slight modifications. The three stages considered were: 1) state A, corresponding to a physiological, fully mobile state, which included animals that could move more than 0.5 cm (freely or upon prodding); 2) state B, representing impaired animals that responded to the prodding, but did not have enough strength to move more than 0.5 cm, and 3) state C, which encompassed animals in a frailty state that barely exhibited head or tail movements or twitches upon prodding.

### Pharyngeal pumping assay

For the pharyngeal pumping assay, separate worm cohorts were cultured, in three independent experiments, each with 60 animals grown on FUdR-supplemented plates seeded with OP50 bacteria. Pumping was monitored and recorded at 1, 5, 10 and 15 days post-L4 moult by filming 13-15 randomly selected worms at each time point. Time lapse recordings were obtained on the Zeiss SteREO Discovery. V20 stereomicroscope (Carl Zeiss AG, Jena, Germany) using the AxioVs40 V4.8.2.0 software (Carl Zeiss AG), using the 1X Plan Apo S objective, at a magnification of 150X; the time lapse captures were converted into.mp4 files using an in-house developed script. Pharyngeal contractions were then accurately counted during a 30-seconds interval. For each strain the average number of pharyngeal movements per minute, standard deviation and standard error of the mean were computed.

### Stress assays and fecundity

Tolerance to heat and oxidative stress was tested for late L4 animals, since responses to both stresses become repressed early in adulthood (starting as early as 4 hours post L4) [89], suggesting that collapse of cellular stress response could represent an early molecular event in the aging process.

### Heat stress assay

To obtain synchronized populations, five hermaphrodites were let to lay eggs for about two hours on three replicate plates. The larvae were reared at 20° C up to late L4 stage, shifted to 35° C for four hours, then returned to 20° C. The percentage of alive animals was scored 48 h later. More than 420 animals were tested for each strain in three individual experiments.

### Oxidative stress assay

Tolerance to oxidative stress was tested by exposure to paraquat and NaN_3_. Both assays were essentially performed as previously described [42]. Briefly, synchronized L4 larvae were transferred to NGM plates containing 2 mM paraquat and survival was scored on day 5 of exposure. For NaN_3_ tolerance, synchronized L4 larvae were collected, washed with M9, incubated for one hour with 0.5 M freshly made NaN_3_ in M9, washed with M9 and placed on a NGM plate to recover. Survival rates were determined after 24 hours.

### Brood size

To analyze the total brood size, L4 worms were placed on NGM plates seeded with OP50 and transferred every 12h to a new plate until they ceased laying eggs. The number of eggs laid by each worm was counted after removal of the parent.

### DAF-16::GFP nuclear translocation

To verify DAF-16 activation we used the CRISPR allele of *daf-16* tagged at the C-terminus with GFP [90]. Since DAF-16 translocation from cytoplasm to nucleus is induced by heat stress and common drugs used to anesthetize the worms [91], the young adult worms were fixed in 4% paraformaldehyde. The worms were grown at 20o C on RNAi plates, at L4 stage 15 μM FUdR was added and next day, the young adults were fixed and immediately visualized. Images were acquired with a Zeiss LSM 710 laser scanning confocal microscope using 10x objective, Argon 488 laser, and identical acquisition setting.

### Statistical analysis

Comparisons between the lifespan values of different strains were carried out by analyzing Kaplan-Meier survival curves. For the statistical analysis and graphical representation of the curves, the R package “survival” was used (https://cran.r-project.org/package=survival).

For the statistical analysis of locomotion, animals were scored by motion stage and plotted as motility curves, similar to the lifespan curves, to evaluate the decline rate of motility in each strain. While in the survival analysis an event represents the death of one worm, in the locomotion analysis an event was defined as the transition between motility states, modeling the population dynamics from increased to low motility (Supplementary Figure 4). Since three motility categories exist, two different motility analyses were conducted, corresponding to two types of events: i) transitions from the mobile state A to either the impaired state B or to the frail state C, and ii) transitions from either state A or B to state C. In the manuscript, only the results from the motility analysis of transitions from A *vs.* cumulated B and C is included, however the two analyses produced very similar results (data not shown).

For all survival and locomotion analyses, the statistical significance was tested using the log-rank test (Mantel-Cox). Comparisons were performed against WT, WT(EV) or *daf-16*, as appropriately. If not otherwise specified, p<0.0001 was considered significant. The p-values were corrected for multiple testing using the Benjamini-Hochberg method, at alpha=0.05.

One-way ANOVA was used for the analysis of pharyngeal pumping, heat, paraquat and sodium azide stress resistance assays. All mutants were compared to wild type and statistical significance was assessed using Dunnett’s test.

## Supplementary Material

Supplementary Figures

Supplementary Table 1

Supplementary Table 2
