# Supplementary material for: Knock-down of odr-3 and ife-2 additively extends lifespan and healthspan in C. elegans
Source: Aging (Albany NY). 2021 Sep 9;13(17):21040–65. doi: 10.18632/aging.203518 (PMC8457566; doi:10.18632/aging.203518)
Supplement: Supplementary Figures [file aging-13-203518-s001.pdf]

SUPPLEMENTARY FIGURES

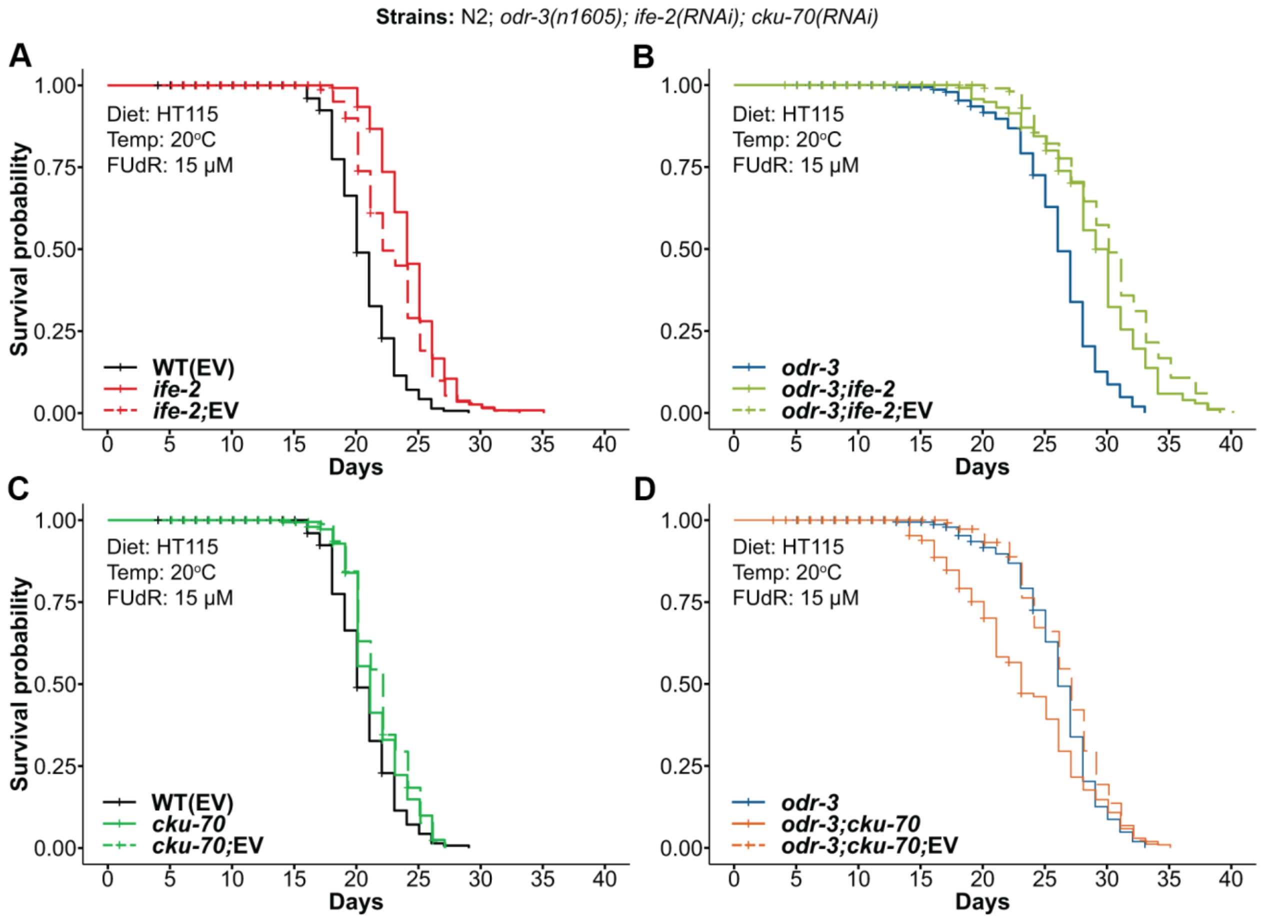

Supplementary Figure 1. Kaplan-Meier survival curves for worms fed bacteria expressing the target dsRNA or an equal mix of target dsRNA and EV. All survival plots represent pooled populations from 3 independent experiments. (**A**, **B**) Lifespan comparison of WT (**A**) or *odr-3(n1605)* (**B**) worms fed bacteria expressing *ife-2* dsRNA or a 1:1 mixture of *ife-2* dsRNA and empty-vector (EV). (**C**, **D**) Lifespan comparison of WT (**C**) or *odr-3(n1605)* (**D**) worms fed bacteria expressing *cku-70* dsRNA or a mixture of *cku-70* dsRNA and EV. Lifespan values are given in Supplementary Table 1.

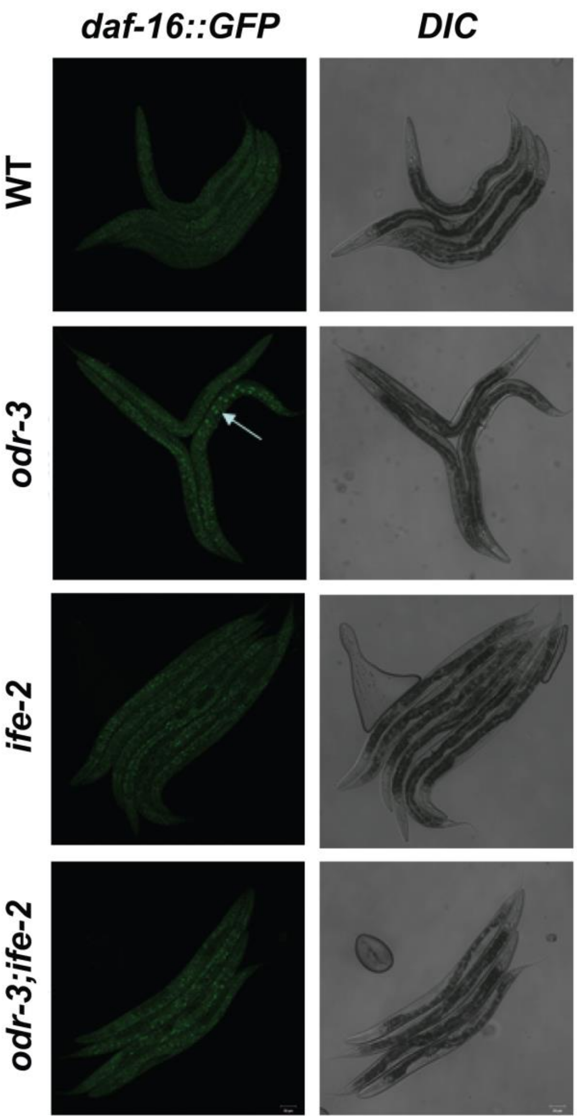

Supplementary Figure 2. DAF-16::GFP nuclear translocation in *odr-3; ife-2 (RNAi)* mutant worms. Worms expressing *daf-16 (ot971 [daf-16::GFP]*) fluorescent marker in WT animals and *odr-3(n1605)*, *ife-2(RNAi)* or *odr-3(n1605); ife-2(RNAi)* mutant background show nuclear accumulation of DAF-16::GFP in *odr-3(n1605)* mutants but not in WT, *ife-2* or *odr-3; ife-2* animals. Left panels show GFP images and right panels show Differential Interference Contrast (DIC) images captured with a confocal microscope. The arrow points to the nuclear accumulation of DAF-16::GFP in intestinal cells of *odr-3* mutant. Images were obtained using the same confocal settings and exposure adjustments were uniformly applied in all images for better visualization.

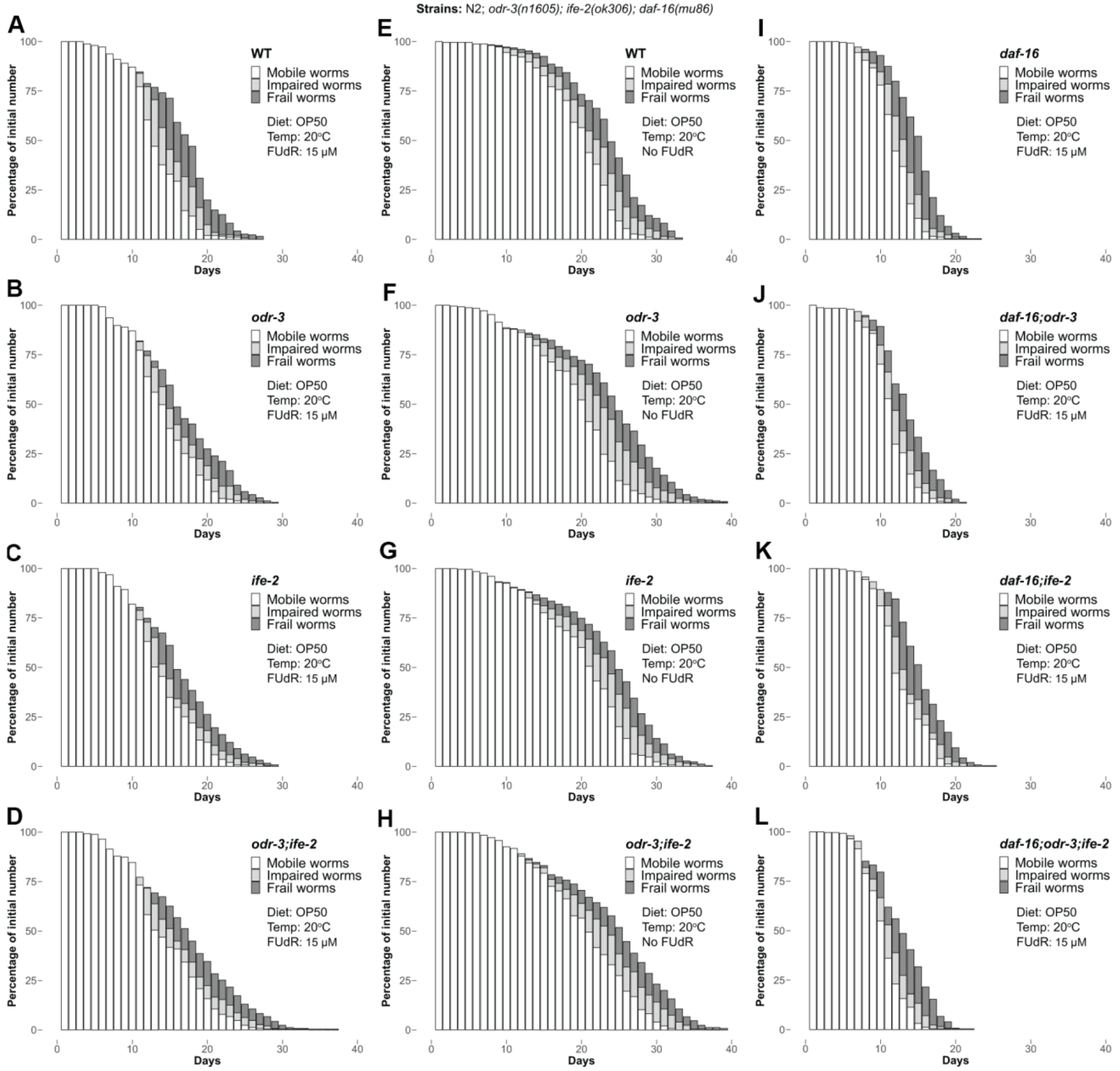

Supplementary Figure 3. Motility-assessed healthspan for mutants containing the *odr-3(n1605)*, *ife-2(ok306)* and *daf-16(mu86)* mutations. (**A**–**L**) Bar chart representation of motility-assessed healthspan illustrating the fraction of each category upon daily monitorization. Worms are grouped into three categories: mobile (white), impaired (light gray) and frail (dark gray). Dead and censored animals were subtracted from these analyses. All cohorts were kept at 20° C and fed OP50 *E. coli*. (**A**–**D**) WT, *odr-3(n1605)*, *ife-2(ok306)* and *odr-3(n1605);ife-2(ok306)* strains on FUdR supplemented plates. (**E**–**H**) WT, *odr-3(n1605)*, *ife-2(ok306)* and *odr-3(n1605);ife-2(ok306)* strains grown on plates with no FUdR. (**I**–**L**) *odr-3(n1605)*, *ife-2(ok306)* and *odr-3(n1605);ife-2(ok306)* in the *daf-16(mu86)* background, grown on FUdR supplemented plates.

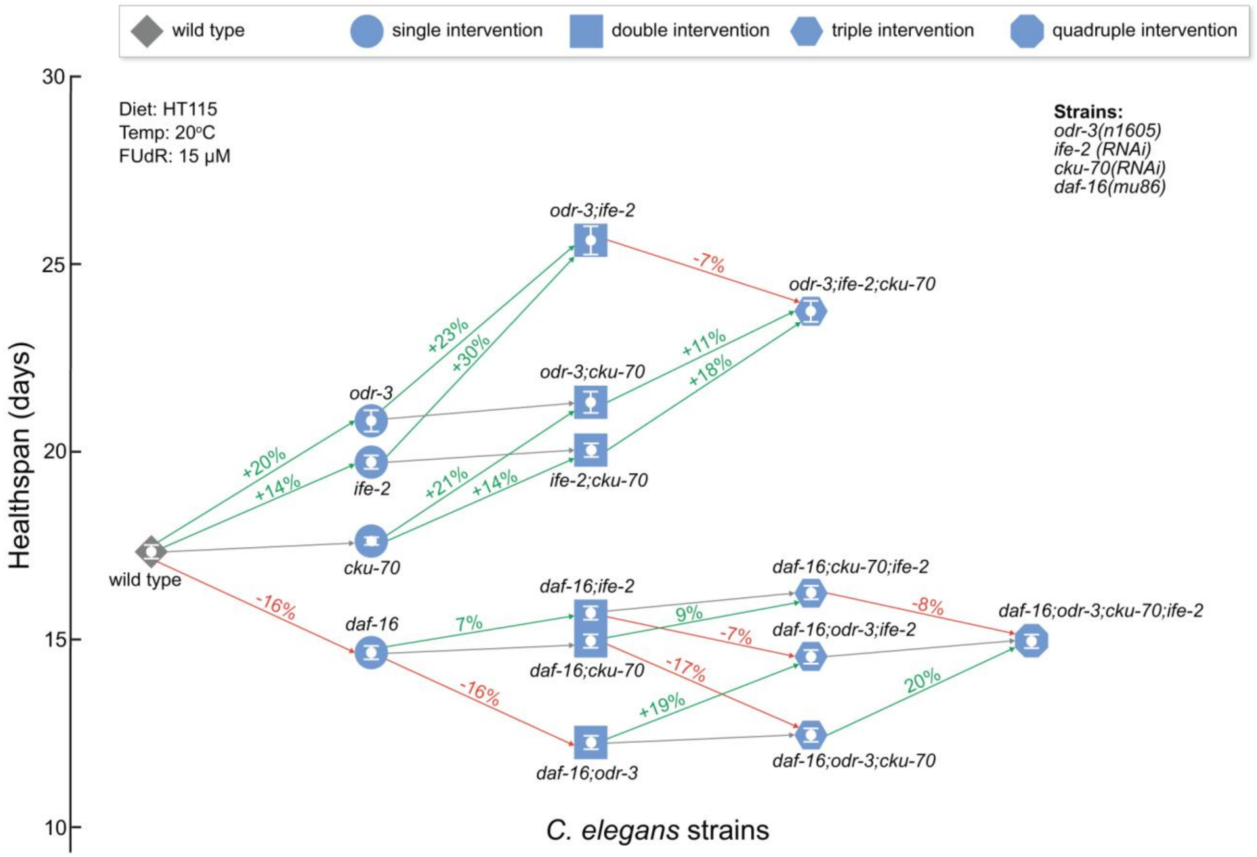

Supplementary Figure 4. Network schematic representation of the strains analyzed in this study and of the effects of each genetic intervention. Nodes represent the strains as follows: diamond for WT, circle for single gene interventions, square for double gene interventions, hexagon for triple gene interventions, and octagon for quadruple gene interventions. Nodes are positioned on the vertical axis according to their respective mean healthspan. Edges between worm strains are colored depending on the gain (or loss) in lifespan extension: increase (green), decrease (red) and small or non-significant change (gray). The extent of the change is included on the edge as a percentage increase/decrease between the origin and destination nodes of the edge. *odr-3* and *daf-16* denote mutants containing the *odr-3(n1605)* and *daf-16(mu86)* mutations; *ife-2* and *cku-70* denote animals in which these genes were modulated by RNAi bacteria. The white bars inside of the nodes indicate the mean ± SEM.

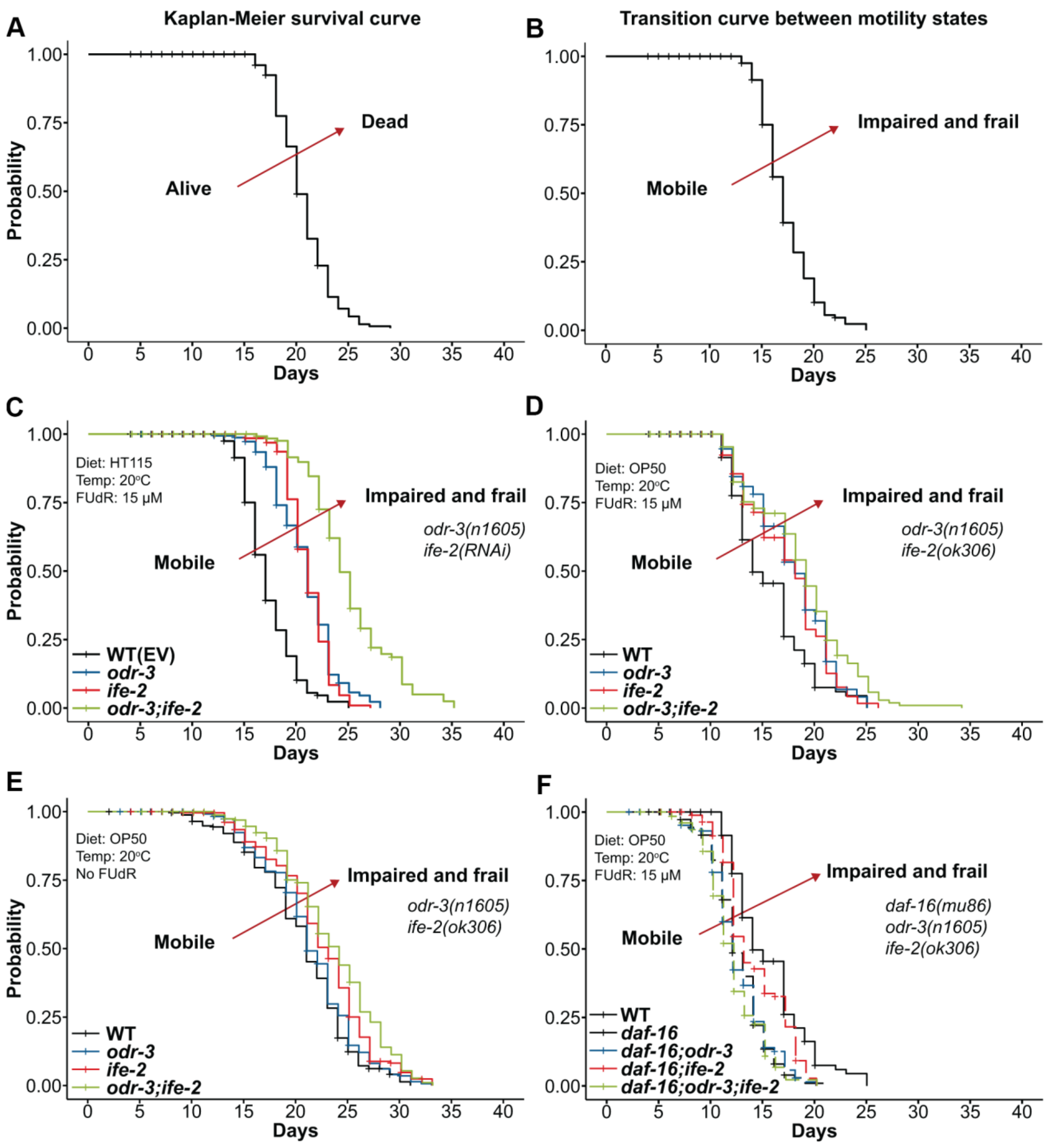

Supplementary Figure 5. Method of quantifying the statistical significance for the healthspan difference between two worm populations. Kaplan Meier curves of survival (**A**) and healthspan (**B**), showing the probability of an event occurring over time. The events represent either the death of a worm (**A**) or the transition from a healthy state to a sick state (becoming impaired or frail) (**B**). For the healthspan analysis, censored and dead worms are removed. (**C**–**F**) Comparison between healthspan curves. Statistical significance was determined using the log rank test.
